# A modified artificial neural network based prediction technique for tropospheric radio refractivity

**DOI:** 10.1371/journal.pone.0192069

**Published:** 2018-03-01

**Authors:** Shumaila Javeed, Khurram Saleem Alimgeer, Wajahat Javed, M. Atif, Mueen Uddin

**Affiliations:** 1 Department of Mathematics, COMSATS Institute of Information Technology, Park Road, Chak Shahzad, Islamabad, Pakistan; 2 Department of Electrical Engineering, COMSATS Institute of Information Technology, Park Road, Chak Shahzad, Islamabad, Pakistan; 3 Department of Physics and Astronomy, College of Science, King Saud University, Riyadh, Saudi Arabia; 4 Department of Information Systems, Faculty of Engineering, Effat University, Jeddah, Saudi Arabia; Southwest University, CHINA

## Abstract

Radio refractivity plays a significant role in the development and design of radio systems for attaining the best level of performance. Refractivity in the troposphere is one of the features affecting electromagnetic waves, and hence the communication system interrupts. In this work, a modified artificial neural network (ANN) based model is applied to predict the refractivity. The suggested ANN model comprises three modules: the data preparation module, the feature selection module, and the forecast module. The first module applies pre-processing to make the data compatible for the feature selection module. The second module discards irrelevant and redundant data from the input set. The third module uses ANN for prediction. The ANN model applies a sigmoid activation function and a multi-variate auto regressive model to update the weights during the training process. In this work, the refractivity is predicted and estimated based on ten years (2002–2011) of meteorological data, such as the temperature, pressure, and humidity, obtained from the Pakistan Meteorological Department (PMD), Islamabad. The refractivity is estimated using the method suggested by the International Telecommunication Union (ITU). The refractivity is predicted for the year 2012 using the database of the previous ten years, with the help of ANN. The ANN model is implemented in MATLAB. Next, the estimated and predicted refractivity levels are validated against each other. The predicted and actual values (PMD data) of the atmospheric parameters agree with each other well, and demonstrate the accuracy of the proposed ANN method. It was further found that all parameters have a strong relationship with refractivity, in particular the temperature and humidity. The refractivity values are higher during the rainy season owing to a strong association with the relative humidity. Therefore, it is important to properly cater the signal communication system during hot and humid weather. Based on the results, the proposed ANN method can be used to develop a refractivity database, which is highly important in a radio communication system.

## 1 Introduction

Radio wave propagations are influenced by the characteristics of the atmosphere and can be scattered, absorbed, reflected, or refracted owing to various atmospheric behaviors. The troposphere is the part of the atmosphere that is closest to human life, and starts from the earth’s surface to a height of about 10 km at the poles and 17 km at the equator. The basic parameters that affect the radio links in the troposphere are the pressure, temperature, and relative humidity [1]. These parameters disturb the frequency and power of a signal. Radio waves have significant importance in radio communications, disaster forecasting, aerospace applications, and environmental monitoring. For example, poor propagations reduce the proper functioning of the communication links and finally bring about a signal decline at the receiver end [2, 3]. The propagation of waves is not limited to the troposphere, and is important to the biomedical fields [4], [5]. Radio refractivity, denoted by *n*, can be defined as the “ratio of radio wave propagation velocity in free space to its velocity in a specified medium” [2]. It can be mathematically written as

$$\begin{array}{c} n=\frac{V_{f}}{V_{m}}. \end{array}$$ (1)

Here, the signal velocity in a free space is denoted as *V*_*f*_, and *V*_*m*_ describes its velocity with respect to a specified medium. In the troposphere, radiowave propagation is evaluated based on variations in the air refractivity [6]. Variations in the refractivity can determine the path of the radio waves. The radio refractive index varies by temperature, humidity, and atmospheric and water vapor pressures. Furthermore, the air temperature, pressure, and humidity also depend on the height at a point above the ground surface. Small variations in these parameters can bring about a substantial effect on the propagation of radio waves, which is due to the fact that the radio signals are refracted over a complete signal path [7]. The refractivity is approximately equal to unity, (i.e., 1.0003) near the earth’s surface [2]. The difference in refractivity from unity depends on two important factors [8]:

- Air is composed of molecules of oxygen, nitrogen, carbon dioxide, and water vapors. These air molecules are polarized when electromagnetic signals pass through them. The interaction between air molecules and electro-magnetic waves is affected by certain atmospheric variables such as the pressure, humidity, and temperature.
- The quantum-mechanical molecular resonance lies within the range of 22 to 60 GHz [8].

The radio refractivity, denoted by *N*, has been used in many studies, and can be expressed mathematically as Radio refractivity denoted by *N*, used in many researches, and can be expressed mathematically as:

$$\begin{array}{c} N=10^{-6}\left(n-1\right) \end{array}$$ (2)

Here, *N* is a dimensionless number and is shown in N units. The radio refractivity relies on the absolute air temperature, *T* (K); vapor pressure, *e* (mbar); and pressure, *p* (mbar). The refractivity *N* can be calculated with the help of the following formula [6, 9].

$$\begin{array}{c} N=\frac{77.6p}{T}+3.75.10^{5}\frac{e}{T^{2}} \end{array}$$ (3)

The water vapor pressure (*e*) can be quantified using the equation given below:

$$\begin{array}{c} e=\frac{RH}{100}a\exp(\frac{bt}{t+c}) \end{array}$$ (4)

where *RH*% represents the relative humidity, *t* and is the temperature (°C). Moreover, the coefficients are given as: *a* = 6.1121, *b* = 17.502, and *c* = 240.97. These coefficients are valid from −20 to 50 °C with an accuracy level ±0.20%.

The refractivity varies based on changes in meteorological parameters [10]. Multiple paths incur larger changes in the atmospheric radio refractive index, for instance, the refractivity is different for different horizontal layers [11]. The same radio wave signals use different paths, and therefore have different arrival times at the target. Moreover, radio signals interfere with each other during their propagation in the troposphere and consequently, the propagation of radio waves makes a curved path towards the earth. Therefore, the refractivity of the atmosphere will change the height as well as the radio signal waves. The performance of a radio signal wave depends on the index of the refractive gradient, which is a function of the atmospheric parameters.

The radio signals in the troposphere are influenced by the changes in meteorological parameters for example, the temperature, pressure, and humidity. These are associated with the changes in weather during different different seasons of the year. These changes in the aforementioned parameters bring about variations in the refractivity [12, 13]. Radiowave propagation is determined by variations in the refractivity of air in the troposphere. Variations in the radio refractivity can curve the path of the propagating radio wave. Moreover, the meteorological parameters are based on altitude. A small variation in any of these parameters can have a significant effect on the radio signals, because they refract over the entire path [14]. The change in refractive index is due to the different phenomena influencing the radio signal, for example, ducting, scintillation, bending, refraction and station interference [2, 11, 15, 16, 17]. Various researchers have studied the effects of meteorological variables on the attenuation of micro or radio waves [18, 19]. It was found that a 10% increase in the temperature and humidity profiles can magnify the attenuation of radio waves by 72.73 × 10^−5^ *dB*/*km* and 2.51 × 10^−2^ *dB*/*km*, respectively. Researchers have indicated that a refractivity database is required for readings observed in air [6, 20, 21]. In general, wireless service providers rely on International Telecommunication Union (ITU) to provide refractive and other relevant databases, owing to the unavailability of accurate local data. These databases are obtained using world meteorological charts and global numerical predictions. Currently, in Pakistan, an accurate local radio refractive database is unavailable. In 2012, S. Ali et al. presented a statistical analysis of the radio refractive index deviations resulting from radio data from 2000 to 2009 for Lahore, Pakistan [8]. In this work, the radio refractivity was calculated with the help of an artificial neural network (ANN) for Islamabad, Pakistan.

ANN models are used to predict a function from the given observations. These are usually applied where unstructured and large data are involved. ANNs can perform different tasks e.g., approximate functions, time series forecasting, data processing, sequence classification, pattern reorganization and numerical control using computers [22–30]. They have many practical fields of application, for instance, system identification and process control, resource management, quantum chemistry, financial applications, medical diagnoses, decision-making, and data mining [31]. Various researchers have already applied ANNs in the prediction of meteorological parameters, [9, 32]. Although the proposed technique was implemented on a smart grid [33], it remains a novel method for the prediction of meteorological parameters and consequently, radio refractivity.

The refractivity changes in the troposphere depends on different factors, and consequently, radio waves effects, such as refraction, bending, and interference from radio stations. The purpose of this research is to analyze meteorological data and develop an efficient tool to predict the effects of temperature, pressure and humidity on a radio link system. To do so, the radio refractivity is predicted. The local meteorological data of Islamabad for the years of 2002 to 2012 were used in calculations of the radio refractivity and prediction. These meteorological data include the temperature, humidity and pressure. The system was trained considering weather data from 2002 to 2011, allowing weather data on the temperature, humidity and pressure to be prediticted for 2012. Afterward, using all predicted values, i.e., temperature, pressure and humidity, the radio refractivity values for 2012 were forecasted. Finally, the predicted refractive values were validated based on the calculated refractive data using real meteorological data for the year 2012.

The objective/importance of this work is to provide a reliable tool for the preparation of a refractivity database. In Pakistan, reliable data at the local level related to atmospheric refractivity are inaccessible. Because the refractivity plays a significant role in a radio communication system, this work presents an ANN method for predicting the different meteorological parameters and radio refractivity.

The remainder of this paper is organized as follows. In Section 2, the proposed ANN model along with the forecast strategies and the data preparation are presented. In addition, data-selection and forecast modules are given to further illustrate the use of artificial neural network. In Section 3, implementation of neural networks for the current problem is described. Next, the results and a discussion are provided in Section 4. In Section 5, some concluding remarks and areas of future research are discussed.

## 2 ANN-based forecast method

Radio refractivity prediction is an application of science and technology. Engineers predict the state of the atmosphere at any given location for the planning and design of a radio link system. They calculate the radio refractivity using meteorological data, i.e., the temperature, pressure and humidity. For the purpose of radio refractivity prediction, we have to predict the weather parameters first. Weather forecasting is one of the most challenging problems globally. The motive behind the current research is to predict the output more accurately. Various prediction strategies are able to handle nonlinearities in the data. ANN can be applied to the forecasting of the nonlinear behaviors. This work employs ANNs to predict the radio refractivity through the use of meteorological parameters, i.e., the temperature, pressure and humidity. The ANN model is implemented in MATLAB. The model was trained using the past ten years of actual data (2002–2011) and validated based on the results of the following year’s data.

The ANN model described herein contains following three modules:

1. Data preparation module
2. Feature selection module
3. Forecast module

The first module is used in pre-processing for the sake of creating compatibility between the input data and the other two modules. The second module is responsible for eliminating unnecessary segments in the data. Finally, the third module is based on an ANN model, and is used to predict the future data.

### 2.1 Data preparation module

As already discussed, the data preparation module receives the input data. Suppose the input data show the following matrix:

$$\begin{array}{c} P=(\begin{array}{ccccc} p_{h_{1}}^{d_{1}} & p_{h_{2}}^{d_{1}} & p_{h_{3}}^{d_{1}} & \cdots & p_{h_{m}}^{d_{1}}\\ p_{h_{1}}^{d_{2}} & p_{h_{2}}^{d_{2}} & p_{h_{3}}^{d_{2}} & \cdots & p_{h_{m}}^{d_{2}}\\ p_{h_{1}}^{d_{3}} & p_{h_{2}}^{d_{3}} & p_{h_{3}}^{d_{3}} & \cdots & p_{h_{m}}^{d_{3}}\\ \vdots & \vdots & \vdots & \ddots & \vdots \\ p_{h_{n}}^{d_{1}} & p_{h_{n}}^{d_{1}} & p_{h_{n}}^{d_{1}} & \cdots & p_{h_{m}}^{d_{n}} \end{array}),\;\;\;\;\; \end{array}$$ (5)

where *h*_*m*_ and *d*_*n*_ denote the days of the *m*^*th*^ month and *n*^*th*^ year, respectively. Moreover, $p_{h_{m}}^{d_{n}}$ represents the historical data.

The value of *m* is equal to the days of the months, and the value of *n* is based on the choice of the designer. When *n* is higher, a finer tuning is applied in the training process (for the third module), which is due to the availability of more samples. However, it will take more time in terms of execution. The data preparation module conducts the following functions.

1. Receives input matrix *p*
2. Computes the local maxima
3. Computes the local normalization
4. Computes the local median
5. Performs the binary encoding
6. Sends the encoded data to the forecast module

Before feeding the ANN using the input matrix *p*, the following functions are executed by the module meant for the data preparation.

1. Local maximum: At the initial stage, the maximum value at each local column of the matrix *p* is computed as follows:
  $$\begin{array}{c} p_{max}^{c_{j}}=max\left\{p_{h_{j}}^{d_{1}},p_{h_{j}}^{d_{1}},p_{h_{j}}^{d_{1}},\cdots ,p_{h_{j}}^{d_{1}}\right\},\forall \;\;j\;\;\epsilon \;\;\left\{1,2,\cdots ,n\right\}. \end{array}$$ (6)
2. Local normalization: The columns of the *p* matrix are normalized based on their respective local maximum value, and the consequent matrix is named as *p*_*nrm*_. The entries of *p*_*nrm*_ range from zero to 1.
3. Local median: The local median value is computed for the columns of the *p*_*nrm*_ matrix, i.e., *med*_*j*_.
4. Binary encoding: A normalized matrix *p* is compared with its respective *med*_*j*_ value. The *med*_*j*_ will be treated as threshold for the values of matrix *P*. The new values of matrix *P* will be considered as “0”if the value of matrix *p*_*nrm*_ is less than corresponding *med*_*j*_ value. Similarly the new values of matrix *P* will be considered as “1”if the value of matrix *p*_*nrm*_ is greater than corresponding *med*_*j*_ value. Now a *P* matrix with binary values is obtained.

### 2.2 Feature selection module

This module applies for the following functions.

1. Removes redundant features
2. Removes irrelevant features

Irrelevant and redundant samples need to be eliminated from the binary encoded data. The execution time of the ANN training process is reduced through the removal of redundant features in the data. This also removes the irrelevant features and improves the forecast accuracy of the network. A mutual-information method based on entropy is applied for the removal of such irrelevant and redundant features [34].

Mutual information between input *L* and target *M* can be calculated using the equation given below:

$$\begin{array}{c} MI\left(L,M\right)=\sum_{i}\sum_{j}P\left(L_{i},M_{j}\right)log_{2}(\frac{P(L_{i},M_{j})}{P\left(L_{i}\right)P\left(M_{j}\right)})\\ \forall \;\;\;i,j\;\;\;\epsilon \;\;\{0,1\}. \end{array}$$ (7)

For a greater value of MI, input *L* and output *M* have a strong relationship. For a lower MI, input *L* and output *M* have a weaker relationship. An MI value of zero indicates that *L* and *M* are not dependent on each other. Thus, the inputs are associated with the MI values between the input and target candidates. Among the training samples, the values are taken as the last samples for every month of the year considered. However, this can propagate serious errors in forecasting because it does not consider the average behavior. We consider both the last sample and the average behavior.

Thus, Eq (7) can be modified for three variables as

$$\begin{array}{c} MI\left(L,M,N\right)=\sum_{i}\sum_{j}\sum_{k}P\left(L_{i},M_{j},N_{k}\right)\times \\ log_{2}(\frac{P(L_{i},M_{j},N_{k})}{P\left(L_{i}\right)P\left(M_{j}\right)P\left(N_{k}\right)})\forall \;\;\;i,j,k\;\;\;\epsilon \;\;\left\{0,1\right\}. \end{array}$$ (8)

For three discrete random samples, we use the eight binary input values given in Table 1.

**Table 1:** Eight binary input values.

*L*	*M*	*N*
0	0	0
0	0	1
0	1	0
0	1	1
1	0	0
1	0	1
1	1	0
1	1	1

Eq (8) can be expanded as

$$\begin{array}{c} MI(L,M,N)=\\ P\left(L=0,M=0,N=0\right)\times log_{2}(\frac{P(L=0,M=0,N=0)}{P(L=0)P(M=0)P(N=0)})\\ +P\left(L=0,M=0,N=1\right)\times log_{2}(\frac{P(L=0,M=0,N=1)}{P(L=0)P(M=0)P(N=1)})\\ +P\left(L=0,M=1,N=0\right)\times log_{2}(\frac{P(L=0,M=1,N=0)}{P(L=0)P(M=1)P(N=0)})\\ +P\left(L=0,M=1,N=1\right)\times log_{2}(\frac{P(L=0,M=1,N=1)}{P(L=0)P(M=1)P(N=1)})\\ +P\left(L=1,M=0,N=0\right)\times log_{2}(\frac{P(L=1,M=0,N=0)}{P(L=1)P(M=0)P(N=0)})\\ +P\left(L=1,M=0,N=1\right)\times log_{2}(\frac{P(L=1,M=0,N=1)}{P(L=1)P(M=0)P(N=1)})\\ +P\left(L=1,M=1,N=0\right)\times log_{2}(\frac{P(L=1,M=1,N=0)}{P(L=1)P(M=1)P(N=0)})\\ +P\left(L=1,M=1,N=1\right)\times log_{2}(\frac{P(L=1,M=1,N=1)}{P(L=1)P(M=1)P(N=1)}) \end{array}$$ (9)

The independent and joint probabilities need to be computed for the MI value between the input and target to determine the joint and independent probabilities. Moreover, we introduce a supplementary variable *B*_*v*_ as

$$\begin{array}{c} B_{v}=4M+2N+L\;\;\;\forall \;\;\;L,M,N\;\;\;\epsilon \;\;\left\{0,1\right\}. \end{array}$$ (10)

It is clear that the range of *B*_*v*_ lies between zero and 7. In addition, *B*_*ov*_, *B*_1*v*_, ⋯,*B*_7*v*_ count the sample points out of a total of *l* points, where *B*_*v*_ = 0, *B*_*v*_ = 1, *B*_*v*_ = 2, *B*_*v*_ = 3, ⋯, and *B*_*v*_ = 7, respectively.

The independent and joint probabilities are given as follows:

$$\begin{array}{c} P\left(L=0,M=0,N=0\right)=\frac{B_{ov}}{l}\\ P\left(L=0,M=0,N=1\right)=\frac{B_{2v}}{l}\\ P\left(L=0,M=1,N=0\right)=\frac{B_{4v}}{l}\\ P\left(L=0,M=1,N=1\right)=\frac{B_{6v}}{l}\\ P\left(L=0,M=0,N=0\right)=\frac{B_{1v}}{l}\\ P\left(L=1,M=0,N=1\right)=\frac{B_{3v}}{l}\\ P\left(L=1,M=1,N=0\right)=\frac{B_{5v}}{l}\\ P\left(L=1,M=1,N=1\right)=\frac{B_{7v}}{l} \end{array}$$ (11)

$$\begin{array}{c} P\left(L=0\right)=\frac{B_{0v}+B_{2v}+B_{4v}+B_{6v}}{l}\\ P\left(L=1\right)=\frac{B_{1v}+B_{3v}+B_{5v}+B_{7v}}{l}\\ P\left(M=0\right)=\frac{B_{0v}+B_{1v}+B_{2v}+B_{3v}}{l}\\ P\left(M=1\right)=\frac{B_{4v}+B_{6v}+B_{5v}+B_{7v}}{l}\\ P\left(N=0\right)=\frac{B_{0v}+B_{1v}+B_{4v}+B_{5v}}{l}\\ P\left(N=0\right)=\frac{B_{2v}+B_{3v}+B_{6v}+B_{7v}}{l} \end{array}$$ (12)

The MI between the input and target is computed and irrelevancy and redundancy are eliminated from the input data points. The MI method has a reasonable execution time and accuracy.

### 2.3 Forecast module

A study of the refractivity and its effects on the troposphere is important because it helps in planning communication links. This study is aimed at an estimation and prediction of the refractivity through the use of meteorological parameters, i.e., the temperature, pressure, and humidity, in Islamabad. The main purpose of the forecast module is to predict the temperature, pressure, and humidity for an estimation of the refractivity. Thus, ANNs are applied by considering the nonlinear characteristics of the weather parameters because an ANN is a powerful modeling technique capable of providing an accurate prediction of the non-linear and complex process of weather forecasting. The data forecast module conducts the following step-wise functions:

1. Receives matrix *P* (binary)
2. Computes the training sample
3. Computes the validation sample
4. Computes the initial output
5. Executes the training process
6. Computes the final output
7. Performs the decoding
8. Performs the de-normalization

The ANN method was chosen for the current study because of the aforesaid reasons. At the initial level, the forecast module gains the selected features, sf. Next, the training samples ts is constructed. Furthermore, validation samples vs is constructed. This relationship is as follows:

$$\begin{array}{c} ts=sf(i,j)\;\;\;\forall \;\;i\;\;\epsilon \;\;\{2,3,\cdots ,m\}\\ \text{and}\;\;\;\forall \;\;j\;\;\epsilon \;\;\{2,3,\cdots ,n\} \end{array}$$ (13)

$$\begin{array}{c} vs=sf(i,j)\;\;\;\forall \;\;j\;\;\epsilon \;\;\{2,3,\cdots ,n\} \end{array}$$ (14)

From Eqs (13) and (14), it is clear that the ANN is trained using all historical data candidates, except the last candidate, which is meant for the purpose of validation. Thus, the training procedure is as previously described, which is based on an ANN. An ANN, which is devised based on a pattern of the human nervous system, comprises a set of artificial neurons to perform different types of tasks (in this study, we want a prediction of the meteorological parameters, i.e., the temperature, pressure, and humidity). In general, artificial neurons apply non-linear mapping ranging from *R*^*I*^ to [0,1] depending on the activation function used.

$$\begin{array}{c} h_{act}^{AN}:R^{I}\to \left[0,1\right] \end{array}$$ (15)

In the above function, *I* = {*I*_1_, *I*_2_⋯*I*_*n*_} is an input signal vector to an AN. To deplete or strengthen an input signal, *I*_*j*_ is linked with weight *R*^*I*^ to *w*_*j*_. The ANN computes I, and also uses $h_{act}^{AN}$ for computing an output signal, *y*. Any bias value *b* (threshold) can influence the strength of *y*. In addition *I* can be computed as follows:

$$\begin{array}{c} I=\sum_{j=1}^{j_{max}}I_{j}w_{j} \end{array}$$ (16)

Here, $h_{act}^{AN}$ is the mapping and obtains *I* and *b* to calculate *y*. Moreover, $h_{act}^{AN}\left(-\infty =0\right)$ and $h_{act}^{AN}\left(+\infty =1\right)$. In addition $h_{act}^{AN}$ uses the sigmoidal, which is given as

$$\begin{array}{c} h_{act}^{AN}\left(I,b\right)=\frac{1}{1+e^{-\alpha (I-b)}} \end{array}$$ (17)

The sigmoid $h_{act}^{AN}\epsilon \left(0,1\right)$ and parameter *α* control the steepness of $h_{act}^{AN}$. Sigmoid $h_{act}^{AN}$ makes AN capable of capturing the non-linear characteristics. Since, this work aims at forecasting of meteorological parameters for the estimation of refractivity. Thus, the ANN consists of separate models for temperature, humidity and pressure using corresponding 2002–2011 values. All of these models predict the temperature, pressure and humidity of the following year. In other words, 36 models are handled individually instead of a single model (because there are 12 months in a year, 12 models are used for the temperature, pressure and humidity, respectively). Moreover, values of the *w*_*j*_ and *b* are determined through learning. In the current study, prior knowledge of the meteorological parameters is available. We supervised the learning by adjusting the *w*_*j*_ and *b* values until the specified termination criteria were no longer fulfilled. The main purpose of supervised training is to adjust the values of *w*_*j*_ and *b* in order to minimize the error signal ‘*ee*(*k*)’ between the target ‘$\hat{z}\left(k\right)$’ and real neuron output ‘*z*(*k*)’ values.

$$\begin{array}{c} \text{Minimize}\;\;\;ee\left(k\right)=z\left(k\right)-\hat{z}\left(k\right)\;\;\;\forall \;\;k\;\;\epsilon \;\;\left\{1,2,3,\cdots ,m\right\} \end{array}$$ (18)

Herein, the multivariate auto regressive model is used for adjusting the weights during the training process [24]. This method is utilized because of its relative accuracy and requires less execution time than other algorithms, for example the gradient descent and Widrow-Holfand delta algorithms [25]. Next, the output matrix is binary decoded, and is de-normalized to obtain the target output.

## 3 Implementation of ANN model

An ANN is generally employed to make a prediction through model training while considering previous datasets. In this work, the proposed ANN model described in Section 2 is employed. Our proposed ANN model consists of neurons, and is organized into three layers: 1) input, 2) hidden, and 3) output layers. The hidden layer is connected to both the input and output layers. Each single node of one layer is partially or fully linked with the nodes in the next layer. The link of the nodes has a particular weight. The value of this weight changes when passing through a connection. A sigmoid activation function is used as a transfer function, and provides the final output at the current node, which may be used as the input for the next node in the next layer. The output layer is always composed of a single node employing a log sigmoid activation function, and produces the predicted results. An error is computed to quantify the difference between the ANN output and the target value (ground truth). The multivariate auto regressive model is applied for adjusting the weights [24]. After adjusting the weights, the entire procedure is repeated using new weights. Adjustments in the weights are also made after every training pattern, and are called a “learning event.” This loop repeats until the error rate becomes stable at a certain acceptable point. Finally, our output layer gives us the final output, which may be treated as a prediction. This ANN architecture has been used to train the given data on the temperature, pressure, and humidity, and has been used to predict the same parameters. Thirty-six such models were formulated to predict the temperature, pressure, and humidity for each month of every year.

Here, the suggested ANN method takes the input parameters, and then multiplies them with the weights and forwards them to a hidden layer. In the hidden layer, the activation function evaluates the data and then forwards the data to the output layer through another activation function, where it finally computes the output of an artificial neuron. In this work, supervised learning is employed, which means the model trains itself using the target values. We provide the target data for every input set. Here, the proposed ANN model obtains the input data using random weights and a sigmoid activation function using hidden layers, and provides the predicted values. Next, the target values are compared with the predicted values, and the error is computed by subtracting these values. Based on this error, the weights are settled again, and the complete process is re-applied for multiple epochs until the error is reduced to the desirable range.

The following procedure was adopted to solve this problem.

1. The network is initialized by setting the weights to random numbers.
2. The input pattern is implemented to obtain the output.
3. The errors for each neuron are computed.
4. An error correction algorithm’ is used to reduce the errors at each iteration.
5. Steps 2 though 4 are repeated to make the target values the actual values.

**Fig 1:**
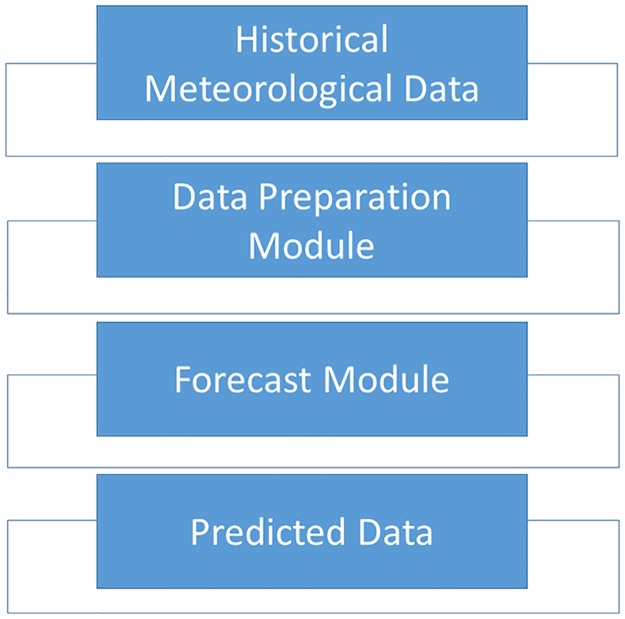
ANN based meteorological forecast.

The radio refractivity is calculated using the predicted values of the temperature, pressure and humidity and then compared with the estimated values of the meteorological data, Fig 1.

## 4 Results & discussions

In this work, the Capital of Pakistan, Islamabad is taken into consideration. The geographical location of Islamabad station is given in Table 2. The RF calculations were made using the local data obtained through the PMD which can be found in the Supplementary Material (S1 File). The objective of this work was to develop a strategy to calculate the RF, which is highly important for a radio wave communication system. In this section, the suggested ANN method is implemented to simulate two different sets of observed values at two different times. The pressure values were observed at the surface level. Moreover, the results are shown date-wise for a two-month period, such as January and August.

**Table 2:** Validation of predicted (ANN) and actual atmospheric values at 00:00 UTC for January 2012.

Date	Actual Temp °*C*	ANN Temp °*C*	RE	Actual Pressure [hPa]	ANN Pressure [hPa]	RE	Actual Humidity %	ANN Humidity %	RE
01	18.97	18.76	0.010	1020.3	1019.2	0.001	94.18	93.14	0.010
02	17.85	17.72	0.007	1020.5	1019.5	0.001	93.90	93.20	0.007
03	17.76	17.65	0.006	1021.5	1020.5	0.001	94.55	93.97	0.006
04	16.47	16.38	0.005	1022.3	1021.2	0.001	94.81	94.32	0.005
05	16.81	16.74	0.004	1020.6	1019.6	0.001	90.90	90.53	0.005
06	17.62	17.55	0.004	1021.6	1020.6	0.001	93.27	92.84	0.004
07	17.47	17.40	0.003	1021.9	1020.9	0.001	94.72	94.33	0.004
08	17.44	17.38	0.003	1022.6	1021.6	0.001	91.72	91.37	0.003
09	17.72	17.66	0.003	1021.4	1020.4	0.001	93.81	93.45	0.003
10	18.59	18.52	0.003	1020.7	1019.7	0.001	92.14	91.64	0.005
11	19.74	19.67	0.003	1021.0	1020.0	0.001	92.45	92.12	0.003
12	19.62	19.56	0.003	1020.2	1019.2	0.001	89.54	89.24	0.003
13	19.90	19.83	0.003	1019.8	1018.8	0.001	91.27	90.96	0.003
14	19.32	19.26	0.003	1020.0	1018.9	0.001	89.18	88.88	0.003
15	18.68	18.62	0.003	1018.3	1017.3	0.001	93.18	92.88	0.003
16	18.11	18.06	0.002	1018.8	1017.8	0.001	91.72	91.43	0.003
17	18.46	18.40	0.003	1017.8	1016.7	0.001	91.45	91.16	0.003
18	17.11	17.06	0.003	1017.9	1016.8	0.001	92.09	91.79	0.003
19	17.51	17.45	0.003	1019.7	1018.6	0.001	91.45	91.16	0.003
20	18.95	18.89	0.002	1019.9	1018.8	0.001	93.63	93.35	0.003
21	18.89	18.83	0.002	1020.1	1019.1	0.001	89.45	89.19	0.002
22	18.01	17.96	0.002	1019.1	1018.1	0.001	92.81	92.54	0.002
23	17.32	17.27	0.003	1018.8	1017.9	0.002	91.36	91.10	0.002
24	18.57	18.52	0.002	1019.7	1018.7	0.001	91.90	91.65	0.002
25	18.80	18.75	0.002	1018.7	1017.7	0.001	92.27	92.01	0.002
26	20.08	20.02	0.002	1019.5	1018.4	0.001	90.81	90.57	0.002
27	20.23	20.17	0.002	1018.6	1017.6	0.001	89.09	88.85	0.002
28	19.19	19.13	0.002	1018.1	1017.1	0.001	90.09	89.85	0.002
29	19.54	19.49	0.002	1019.3	1018.3	0.001	91.18	90.94	0.002
30	19.29	19.23	0.002	1019.5	1018.5	0.008	91.72	91.49	0.002
31	18.85	18.75	0.005	1019.9	1018.9	0.001	88.45	88.23	0.002

### 4.1 Test problem 1

**Fig 2:**
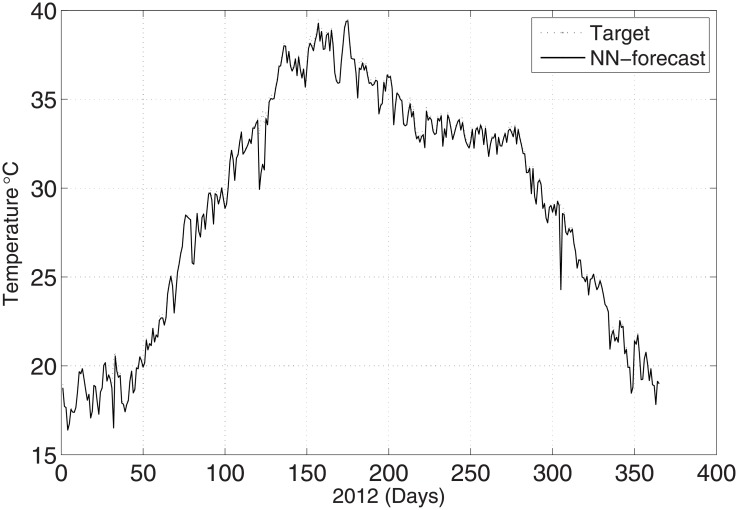
Test problem 1: Actual and predicted (ANN) temperature values.

To validate the predicted and actual results, the meteorological parameters were observed at 00:00 UTC with the maximum temperature. Fig 2 shows the prediction of temperature for the year 2012 when employing the ANN method. The results are compared with the real-time data for the same year. Fig 2 shows the good agreement between the predicted and original data. The relative error is calculated with the help of the following formula:

$$\begin{array}{c} \text{Relative}\;\text{Error}=RE=\frac{\left|\text{actual}\;\text{values-predicted}\;\text{values}\right|}{\left|\text{actual}\;\text{values}\right|} \end{array}$$ (19)

**Fig 3:**
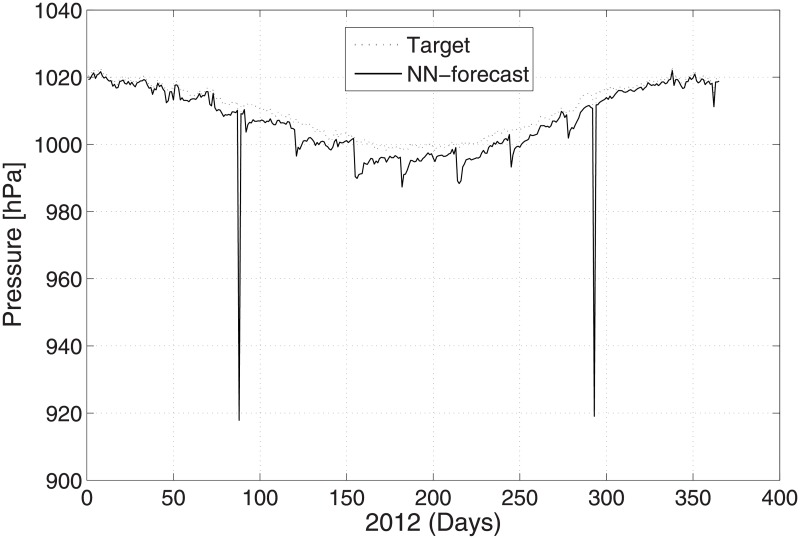
Test problem 1: Actual and predicted (ANN) pressure values.

**Fig 4:**
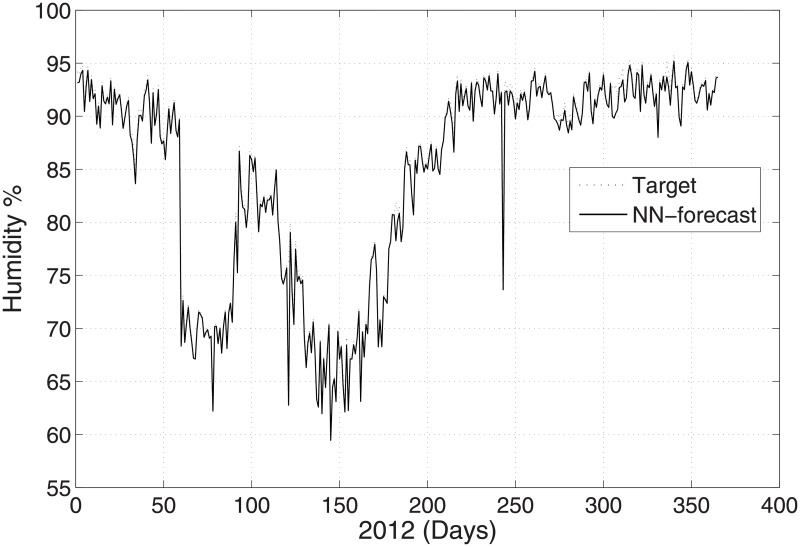
Test problem 1: Actual and predicted (ANN) humidity values.

**Fig 5:**
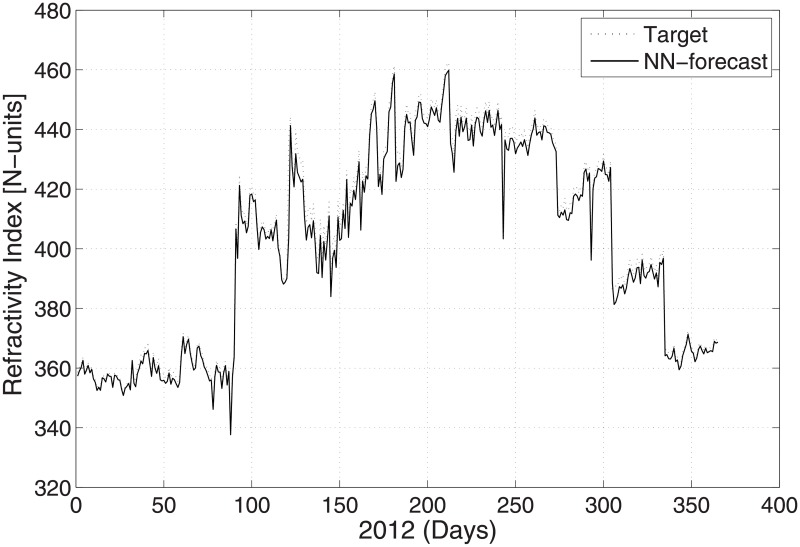
Test problem 1: Actual and predicted (ANN) refractivity values.

The predicted and original/actual values of the atmospheric parameters and relative errors for January are presented in Table 3. The RE is less than 0.5%, which shows the high accuracy of the proposed ANN method. The comparison between the predicted and actual values for pressure is shown in Fig 3. The predicted and original values for pressure agree well with each other even for the sharp changes at around day 85 and day 290. The predicted and actual relative humidity values are closer to each other, as presented in Fig 4. Finally, the refractivity is computed using the predicted values of temperature, pressure, and humidity. The comparison between the predicted refractivity and target refractivity is given in Fig 5. Next, the validation between the actual and predicted refractivity is given in tabulated form, as shown in Table 3. The error is again less than 0.5%, which guarantees the accuracy of the suggested ANN algorithm. One can expect more error in the refractivity as the errors in the temperature, pressure, and humidity accumulate, as can be seen in Eq (3).

**Table 3:** Validation of predicted (ANN) and actual refractivity at 00:00 UTC for January 2012.

Date	Original Refractivity [N-units]	predicted Refractivity [N-units]	Relative Error
01	358.5966558	357.3032355	0.0036
02	360.1198256	359.0117554	0.0031
03	361.1280077	360.1018744	0.0028
04	363.5921161	362.5986368	0.0027
05	358.9210021	357.9507057	0.0027
06	360.1786946	359.2377162	0.0026
07	361.8631692	360.9376305	0.0025
08	359.2642328	358.3820866	0.0024
09	360.4683403	359.5742551	0.0024
10	357.2654745	356.3930749	0.0024
11	356.0299676	355.1891191	0.0023
12	353.2988769	352.4948633	0.0022
13	354.3691739	353.5564918	0.0022
14	353.3391614	352.5285757	0.0022
15	357.5902174	356.7624538	0.0023
16	357.2307459	356.4020746	0.0023
17	356.1661665	355.3428191	0.0023
18	358.8403278	357.9958653	0.0023
19	358.1381129	357.2975487	0.0023
20	358.0134116	357.1850137	0.0023
21	354.2832295	353.4926109	0.0022
22	358.4740361	357.6543881	0.0022
23	358.1151224	357.3088668	0.0022
24	356.9399129	356.1410981	0.0022
25	356.6639553	355.8651627	0.0022
26	353.5927648	352.8227771	0.0021
27	351.5539546	350.7959796	0.0021
28	353.8966248	353.1282681	0.0021
29	354.6855124	353.9132697	0.0021
30	355.6244574	354.8509198	0.0021
31	353.4258512	352.6652588	0.0021

The refractivity is less from January to June, but increases from July to August, and after which starts declining. This is due to the fact that the high refractivity can be expected in the rainy season because the relative humidity is high. The correlation coefficients of the radio refractivity with the considered meteorological parameters, temperature, pressure, and relative humidity, in Islamabad are 0.72, 0.56, and 0.87 respectively. In August, the correlation coefficients are 0.45, 0.15, and 0.98 respectively. These coefficients demonstrate that all of the parameters have a strong relationship with the refractivity, particularly the temperature and humidity. The results further indicate that the relative humidity has greater effects on the refractivity than the other two parameters for both months and seasons. Moreover, the relative humidity has a significant influence on the refractivity during the rainy month of August.

The actual and predicted values of refractivity for August 2012 are shown in Fig 5. literature survey shows that higher refractivity lowers the signal strength and vice versa [35]. A high refractivity has an effect on the radio signal, and consequently, the wireless communication system may not function properly. The data is taken from geographical location of PMD with latitude of 33.68^*o*^ N, longitude of 73.06^*o*^ E and Altitude of 540 meters.

### 4.2 Test problem 2

**Fig 6:**
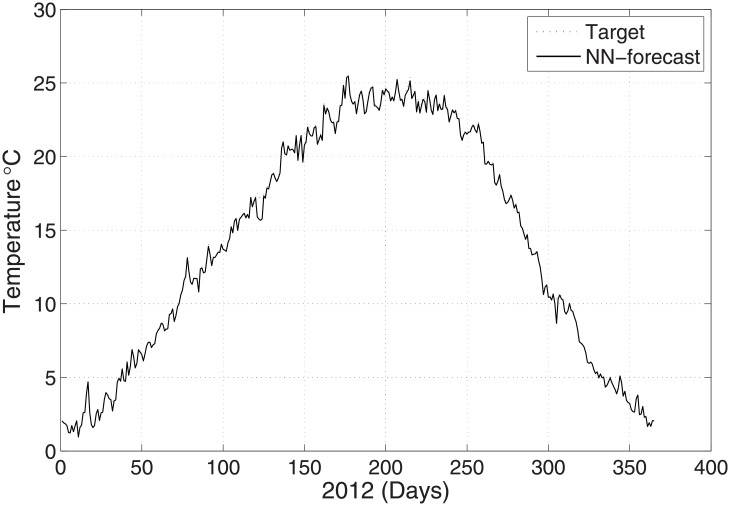
Test problem 2: Actual and predicted (ANN) temperature values.

**Fig 7:**
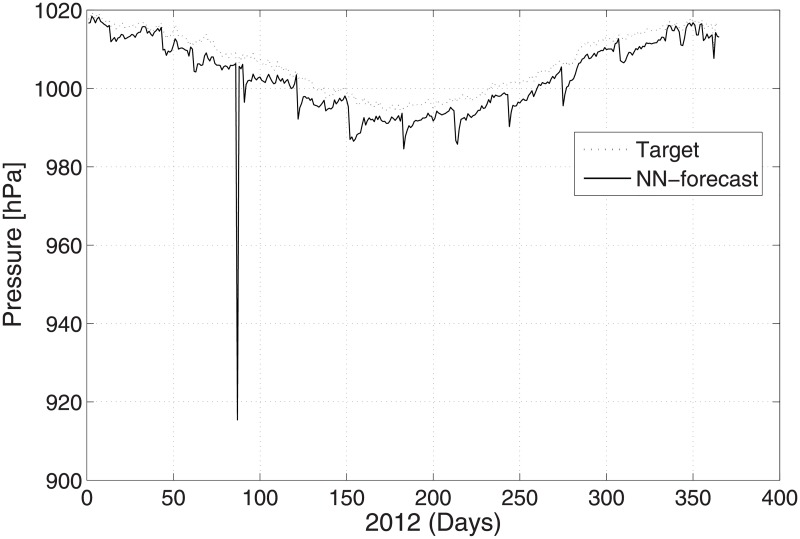
Test problem 2: Actual and predicted (ANN) pressure values.

**Fig 8:**
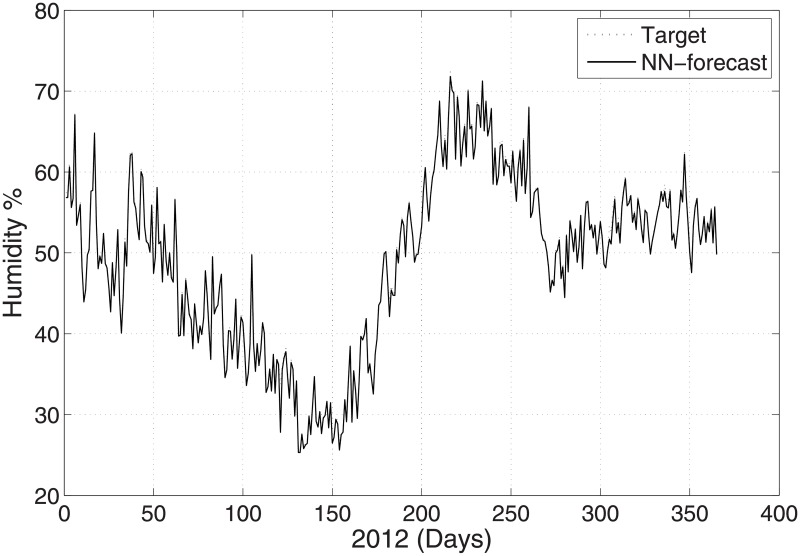
Test problem 2: Actual and predicted (ANN) humidity values.

**Fig 9:**
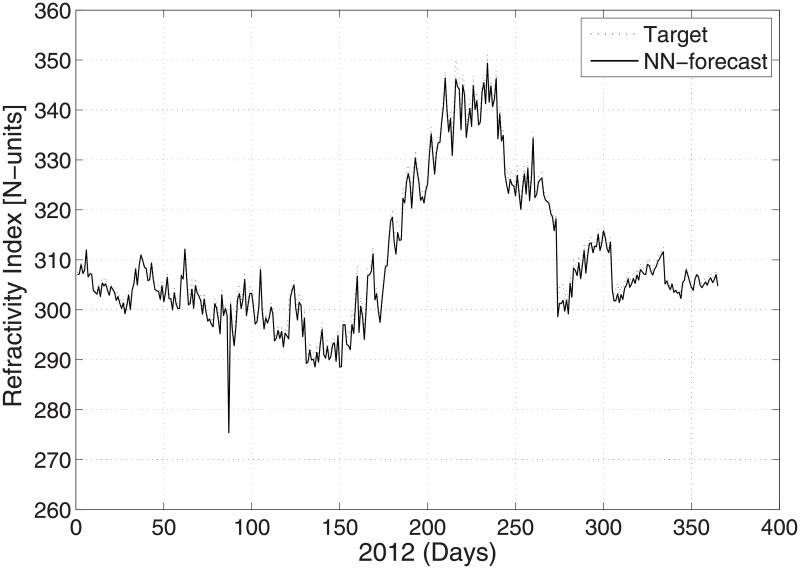
Test problem 2: Actual and predicted (ANN) refractivity values.

In this subsection, prediction of the meteorological values of the temperature, pressure, and humidity observed at time 12:00 UTC with the minimum temperature is described. Fig 6 shows the temperature prediction for the year 2012 when employing the suggested ANN method. The results are validated against the real time PMD data for the same year. The validation between the predicted and actual values for the pressure and humidity is shown in Figs 7 and 8, respectively. The figures show that the predicted and actual values are close to each other. Moreover, the refractivity is estimated using the atmospheric database obtained using PMD. Next, the predicted and estimated values of refractivity are calculated, and as demonstrated in Fig 9, which shows a good agreement between the predicted and actual refractivity, thereby illustrating the accuracy of the proposed method. The refractivity with an error is also performed and found to be less than 0.5% for January 2012. The error less than 0.5%, verifies strengths argument regarding the employment of the ANN algorithm. As the values observed for 12:00 UTC indicate, the refractivity database trend shows a smaller value from January to June, but increases from July to August, after which it starts declining. This is due to the fact that a high refractivity can be expected during the rainy season. The refractivity for the month of August is calculated and the RE value found to be insignificant which shows the validity of the proposed method. It is worth dealing with a high refractivity to achieve a proper functioning of a communication system.

## 5 Conclusion

Radio refractivity changes in the troposphere are important feature influencing electromagnetic waves, and finally communication systems. The refractivity varies through changes in the meteorological parameters. The focus of this work was to provide a reliable tool for preparing a refractivity database. In Pakistan, local reliable data related to atmospheric radio refractivity are unavailable and the refractivity is important in the planning and design of radio links. In this work, ANN models that consider the temperature, pressure, and humidity are used to calculate the refractivity. Meteorological data of eleven years from 2002 to 2012 were obtained from PMD, Islamabad. Ten years of data from 2002 to 2011 was used for training the system and data of 2012 was used to verify the results with real time values of refractivity index. The refractivity was estimated using a method suggested by ITU. The refractivity was predicted using ANNs and implemented in MATLAB. Next, the refractivity predicted whenemploying ANNs was compared with the estimated refractivity obtained using the PMD database. The predicted and original/actual values of the atmospheric parameters agree well and demonstrate the accuracy of the proposed ANN method even for the sharp changes in humidity, pressure, and refractivity. The estimated and predicted refractivity was then validated. Moreover, the RE was found to be less than 0.5% for all cases.

The refractivity values were less from January to June and September to December, whereas higher values were observed from July to August. This is due to the fact that a high refractivity can be expected in the rainy season because the relative humidity is high. The correlation results of refractivity with the considered meteorological parameters demonstrate that all of the parameters have a strong relationship with the refractivity, particularly the temperature and humidity. Furthermore, the relative humidity has a significant effect on the refractivity as compared to the other two parameters for both months, particularly during the rainy season. Therefore, it is important to properly take care of a signal communication system during hot and humid weather.

Based on the results obtained in this study, it can be concluded that the proposed ANN method can be used for an estimation of the humidity, pressure, and refractivity. In general, the proposed algorithm can be used for all types of relevant weather analysis.

## Supporting information

S1 File Supplementary material. Daily average value of temperature, pressure, and humidity from 2002 to 2012. https://doi.org/21.1/journal.pone.2018.s0.
(XLSX)
